# Ethiopians' knowledge of and attitudes toward epilepsy: A systematic review and meta-analysis

**DOI:** 10.3389/fneur.2023.1086622

**Published:** 2023-02-28

**Authors:** Beshada Zerfu Woldegeorgis, Eyosiyas Abreham Anjajo, Tesfalem Israel Korga, Berhanu Lijalem Yigezu, Efa Ambaw Bogino, Habtamu Tieka Tema, Henok Berhanu Alemu, Tesfalem Israel Boda, Dugo Angasa Daba, Negeso Gobena, Mohammed Suleiman Obsa

**Affiliations:** ^1^Department of Internal Medicine, Wolaita Sodo University, Wolaita Sodo, Ethiopia; ^2^Department of Pathology, Wolaita Sodo University, Wolaita Sodo, Ethiopia; ^3^Department of Obstetrics and Gynecology, Wolaita Sodo University, Wolaita Sodo, Ethiopia; ^4^Department of Dermatovenereology, Wolaita Sodo University, Wolaita Sodo, Ethiopia; ^5^Department of Surgery, Wolaita Sodo University, Wolaita Sodo, Ethiopia; ^6^Department of Anesthesia, Hawasa University, Hawassa, Ethiopia; ^7^Department of Anesthesia, Arsi University, Assela, Ethiopia

**Keywords:** attitudes, awareness, epilepsy, Ethiopia, knowledge

## Abstract

**Background:**

Epilepsy remains one of the world's most common neurological diseases, but it appears to be widely misunderstood, particularly in under-resourced countries like Ethiopia. Improving individuals' knowledge and attitude toward epilepsy is critical for reducing the multifaceted impacts of epilepsy. Therefore, in this study, we sought to estimate the pooled levels of good knowledge and a favorable attitude toward epilepsy and also identify the associated factors using available data collected from different segments of the population.

**Methods:**

Articles were searched in international electronic databases. A standardized Microsoft Excel spreadsheet and STATA software version 16 were used for data extraction and analysis, respectively. The Preferred Reporting Items for Systematic Reviews and Meta-Analysis (PRISMA) checklist was used to write this report. The random-effect meta-analysis model was used to estimate Der Simonian-Laird's pooled effect. Statistical heterogeneity of the meta-analysis was checked *via* Higgins and Thompson's *I*^2^ statistics (0–100%), and Cochran's *Q* test at *P* < 0.10. Subgroups, based on the study regions, and sensitivity analyses were also performed. Publication bias was examined subjectively using funnel plots and objectively using the nonparametric rank correlation test of Begg and the regression-based test of Egger for small study effects with *P* < 0.05 considered to indicate potential publication bias. Furthermore, the Trim-and-fill method of Duval and Tweedie was used to explore sources of publication bias for the favorable level of attitudes toward epilepsy.

**Result:**

A total of 12 studies with 6,373 study participants and 10 studies with 5,336 study participants were included to estimate the pooled level of good epilepsy knowledge and favorable attitudes respectively. The overall estimated levels of good epilepsy knowledge and favorable attitudes toward epilepsy among Ethiopians were 47.37% [(95% CI: 35.00, 59.74), *I*^2^ = 99.2, *P* < 0.001] and 46.83%[(95% CI: 32.75, 60.90), *I*^2^ = 99.2, *P* < 0.001] respectively. Subgroup analysis revealed that the pooled level of good epilepsy knowledge was 48.51% [(95% CI: 38.95, 58.06), *I*^2^ = 95.6%, *P* < 0.001] in the Amhara region.

**Conclusion:**

In the current review, we found out that there is a huge knowledge gap and an unfavorable level of attitudes towardepilepsy, which demand immediate public health action as well as a targeted policy intervention.

## Background

Epilepsy is defined as at least two unprovoked seizures occurring in a time frame of more than 24 h apart. It is also considered present when the recurrence rate of a single unprovoked seizure is more than 60% over the next 10 years, or when a diagnosis of epilepsy syndrome is made (1, 2).

Epilepsy affects people of all races, social classes, national boundaries, and all ages, but has a bimodal distribution with the highest risk in the youngest and oldest age groups (3). To date, over 70 million people have been affected by epilepsy, and it is responsible for about 1% of the global burden of disease (4). As a result, epilepsy has grown to be a major health concern (5). Furthermore, due to the higher incidence of symptomatic epilepsy following, for instance, birth asphyxia, cerebral malaria, other central nervous system infections, head trauma, birth injury and so on, low and middle-income countries constitute ~80% of the global incidence (80–100 per 100,000 people per year) of epilepsy, where the condition remains largely untreated (6).

The estimated proportion of the general population with active epilepsy (i.e continuing seizures or with the need for treatment) was estimated to be 32.7 million people worldwide (7). Besides, the cumulative lifetime incidence of epilepsy is 3% and more than half of the disorders start in childhood. The annual prevalence is 0.5–1% (8), meaning it is an unrecognized and underreported public health problem around the world (7). Over 85% of people with the disease do not start treatment, and ~90% of those who are untreated are unaware that epilepsy treatments exist (9).

A meta-analysis of a door-to-door population-based survey involving 1,137,491 people in Sub-Saharan Africa revealed that 16 per 1,000 people had active epilepsy, with only modest variations between regions (10). Despite the scarcity of epilepsy research in Ethiopia, a door-to-door survey in Zay villages (in 2006), Oromia region, found a high prevalence rate of active epilepsy of 29.5 per 1,000 people (11).A similar study in central Ethiopia (1986–1988), involving 60,820 inhabitants, reported an active epilepsy prevalence of 5.2 per 1,000 people (12).

The jeopardy of epilepsy is multidimensional and grave; the onset of seizures is usually explosive and unpredictable, imposing a substantial risk of physical injury, hospitalization, and death, and negatively inflicting a patient's mental health, often resulting in anxiety, depression, or cognitive impairment, stigmatization and its social and economic depression (5, 13).

Religious and sociocultural beliefs have detrimental effects on the type of epilepsy care and treatment that people with epilepsy (PWE) should receive. Many African communities associate epilepsy with evil spirits and superstitions, urging traditional healers, fey priests, and religious leaders to treat them (14, 15). Although epilepsy is a common disorder and there are highly effective and low-cost treatment options, the disease is widely misunderstood by others and the patients themselves (5). Thus, in developing countries, 60–90% of PWE do not receive treatment (16).

The level of knowledge and exhibited attitudes of individuals have an impact on epilepsy stigma (17). A national wide survey conducted in Italy pointed out that 93.4% of the population had good knowledge of epilepsy (18).

Uslu et al. (17) reported hospital staff had moderate knowledge and a favorable attitude, in a hospital located on the eastern side of Turkey. In a systematic review by Jones et al. (19), it was underscored that teachers in all parts of the world where they had been studied had inadequate knowledge of epilepsy and negative attitudes toward epilepsy. Moreover, studies in the Kuwaiti population demonstrated that ~97.6% of sampled population had good knowledge, but a far more negative attitude toward epilepsy (20, 21).

People with epilepsy have been denied necessary care and assistance due to a lack of adequate understanding and favorable attitudes among the general public. Improving the general public's knowledge and attitude toward epilepsy, as well as that of schoolteachers and students, epilepsy patients, relatives of PWE, and healthcare workers is critical for reducing stigma, drug adherence problems, withdrawal from school among students with epilepsy, and other multifaceted impacts of epilepsy. However, existing primary studies of knowledge and attitude toward epilepsy in Ethiopia have reported very discrepant and inconsistent results, which call for a growing demand to conduct systematic reviews and meta-analyses.

Therefore, the current review aimed to show the pooled estimate for the level of good knowledge and favorable attitude toward epilepsy in Ethiopia and identify the associated factors. The findings of the current review will hopefully serve as a springboard for large-scale community and institutional-based educational intervention packages focusing on different segments of the population. This is certainly relevant in Ethiopia, where the burden of epilepsy is brisk.

## Methods

### Study protocol registration

This systematic review and meta-analysis was undertaken to estimate the level of good knowledge of and favorable attitudes toward epilepsy among Ethiopians and to identify the associated factors. The study protocol for this review was registered in an international database, the Prospective Register of Systematic Reviews (PROSPERO), by the University of York Center for Reviews and Dissemination (CRD), on May 10, 2022 (**https://www.crd.york.ac.uk/prospero/display_record.php**, identifier: CRD42022327872) to promote and maintain transparency in the systematic review process, minimize the risk of reporting bias, and reduce unnecessary review duplication. Furthermore, while the review was in progress, a protocol amendment was made (July 2, 2022) regarding the title, review stages, and completion dates of the review, and records were submitted online to the CRD editorial team. A 17-item Preferred Reporting Items for Systematic Reviews and Meta-Analyses Protocols (PRISMA-P) 2015 checklist was used to guide protocol development (22).

### Reporting

The Preferred Reporting Items for Systematic Reviews and Meta-Analyses (PRISMA) 2020 Checklist was used to report the review's findings (23) (Supplementary Table 1).

### Inclusion criteria

The inclusion criteria for this review were based on the study characteristics and report characteristics determined by using the CoCoPop (condition, context, and population) mnemonic (24). Thus, we included all observational studies (cross-sectional studies, case-control studies, and cohort studies). **Participants/Population**: The general public, school teachers, students, people with epilepsy, healthcare workers, relatives, or families of PWE who participated in the studies that assessed the level of knowledge and attitudes toward epilepsy and/or associated factors were considered. **Context**: Limited to primary studies conducted in Ethiopia. **Language of publication**: Articles reported in English were included. **Years of publication**: Articles published between 2010 and 2022 were included.

### Exclusion criteria

Studies without full-text access; articles that contained insufficient information; findings from personal opinions; articles reported outside the scope of the outcome of interest; qualitative study design; case reports; case series; letters; unpublished data; and previous systematic reviews were filtered out.

### Information sources and search strategy

Literature search strategies were developed using medical subject headings (MeSH) and text words related to the outcomes of the study. The search typically included the following electronic bibliographic databases: Excerpta Medica database, PubMed, Web of Science, African Journal of Online, Google Scholar, and Cochrane Library to ensure complete coverage of the topic by accounting for variability between the indexing in each database. The literature search was limited to studies published in the English language between 1^st^ January 2010 and February 30^th^, 2022 which explored epilepsy knowledge, attitudes, and/or associated factors among Ethiopians. The reference lists of included studies identified through the search were scanned to ensure literature saturation. Where necessary, we also searched the authors' files to ensure that all relevant materials had been captured. For the advanced search in PubMed, the following steps comprised the search process: Initially, the search statement was divided into four main concepts: epilepsy, knowledge, attitude, and Ethiopia. Subsequently, we gathered keywords from Google scholar, Wikipedia, and Google for each concept, which was then searched independently in PubMed to find MeSH terms in the MeSH hierarchy tree and then combined in an advanced search. Boolean operators (AND and OR) were used to combine these four concepts as follows: (((Knowledge) OR (“Knowledge” [Mesh]))) AND ((“Attitude” [text word]) OR (“Attitude” [MeSH Terms])) AND ((“Ethiopia” [Mesh]) OR (Ethiopia^*^ (text word))). Finally, we filtered the results to include just the most relevant ones. The search was double-blinded and conducted from February 30^th^ to April 20, 2022, by two authors (BW and MO). A separate file with the search details was supplied (Supplementary Table 2).

### Study selection procedures

The articles that were found through the electronic database searches were exported to the reference management software, Zotero, where duplicate studies were then eliminated. Two authors (BW and MO) independently screened the titles and abstracts that were obtained by the search against the inclusion criteria. To describe the extent to which assessments by multiple authors are similar, inter-rater agreement was calculated after referring to the Cochrane handbook for systematic reviews. In this case, a kappa value of 0.75 and above was considered, indicating excellent agreement. The screened articles were then subjected to a full article review by two independent authors (NG and EA). A pre-defined eligibility criterion was used to determine which records were relevant and should be included in the review. Where more information was required to answer queries regarding eligibility, the remaining authors were involved. Disagreements were resolved through discussion. Moreover, the reasons for excluding the articles were recorded at each step.

### Data extraction

Two authors (**BW and MO**) abstracted the relevant data independently by using a standardized Microsoft Excel spreadsheet. For data extraction, JBI-adopted formats were employed (25). The first author's name, sample characteristics, regions of study, year of publication, study design, study area, outcome measures, timing and procedures of data collection, response rates, knowledge of and attitudes toward epilepsy were collected. The reliability agreement among the data extractors was evaluated and verified using Cohan's kappa coefficient after data was recovered from 30 percent of the primary studies. As a consequence, the kappa coefficient's strength of agreement was divided into four categories: low (0.20), fair (0.21–0.40), moderate (0.41–0.60), good (0.61–0.80), and virtually perfect agreement (0.81–1) (26). A kappa statistic value of more than or equal to 0.5 was regarded as congruent and acceptable. In the case of disagreements between the two data extractors, a third author (**EA)** was involved in adjudicating unresolved disagreements through discussion and re-checking of the original articles.

### Study definition and outcome measurement

In terms of good epilepsy knowledge, we calculated the point estimate after directly taking the absolute number of participants who were reported by the authors of the primary studies as having an adequate or good level of knowledge based on the “yes” or “no” response (27–29), and scoring mean and above (30–37). Similarly, we computed the point estimate of favorable attitudes from the absolute frequencies of participants found to have favorable attitudes based on the mean and above score (27–36) considering the existence of some heterogeneity in the operationalization of the outcome. Finally, we determined that a score of 50% and above indicated good knowledge and positive attitudes.

Furthermore, associated factors were narrated in texts as socio-demographic and other related characteristics as we identified insufficient data on factors influencing Ethiopians' knowledge of and attitudes toward epilepsy to conduct the meta-analysis, and the included primary studies that assessed the determinant factors had heterogeneous explanatory variables classification concerning the outcome variables.

### Methodological quality (risk of bias) assessment

To assess the quality of the studies, the Joana Briggs Institute (JBI) critical appraisal checklists (38) for cross-sectional study (analytical or descriptive) were employed. Three authors (**TK, NG, and BY**) independently assessed the methodological quality of each study. In this manner, the following components were evaluated for studies reporting purely descriptive cross-sectional data: appropriateness of the sample frame for addressing the target population, sample size adequacy, study setting, and participants, and whether the data analysis was conducted with sufficient coverage of the identified sample, validity and reliability of the measurement, appropriateness of the statistical analysis, and adequacy and management of response (Supplementary Table 3). In addition, the JBI checklist assessed the following main components for the analytical cross-sectional studies: inclusion criteria, participants and settings, whether the exposure and outcome were measured validly and reliably, whether standard and objective criteria were used for measuring the outcome, confounding factors, and strategies used to deal with them, and the appropriateness of the statistical analysis (Supplementary Table 4). Disagreements were resolved through consultation with a third independent reviewer (**EB**). Studies with a score of 7 or higher after being evaluated against these criteria were considered low risk and included in this systematic review and meta-analysis.

### Data synthesis and meta-analysis

The extracted data were imported from a Microsoft Excel spreadsheet into STATA MP 16 statistical software (StataCorp LP, 4905 Lakeway Drive, College Station, TX 7845, USA) for analysis. The heterogeneity of the results was visually examined *via* the forest plots with pooled estimates. Thus, its presence was confirmed subjectively with a lack of overlap between the confidence interval (CI).In addition, the statistical heterogeneity was explored more formally by using Cochran's *Q*-test (*x*^2^) at *P*-value < 0.10 indicating significant heterogeneity. Another heterogeneity measure, Higgins and Thompson's I^2^ statistics, was employed to estimate the percentages of the between-study variability where, 0, 25–50, 50–75, and ≥75% indicated no heterogeneity, low heterogeneity, moderate heterogeneity, and high heterogeneity respectively (22). The random-effect meta-analysis model was used to estimate Der Simonian and Laird's pooled effect due to the presence of considerable statistical heterogeneity. Subgroup meta-analysis based on the study regions as covariates, meta-regression, and sensitivity analyses were also performed to investigate the source of statistical heterogeneity. Publication or dissemination bias was examined subjectively using funnel plots and objectively using the non-parametric rank correlation test of Begg (39) and the regression-based test of Egger for small study effects (40), with *P* < 0.05 being taken into consideration to declare potential publication bias. In the presence of publication bias, the non-parametric trim-and-fill method of Duval and Tweedie was conducted. Results were presented in the form of tables, texts, and figures.

## Result

### Search and study selection

**Figure 1:**
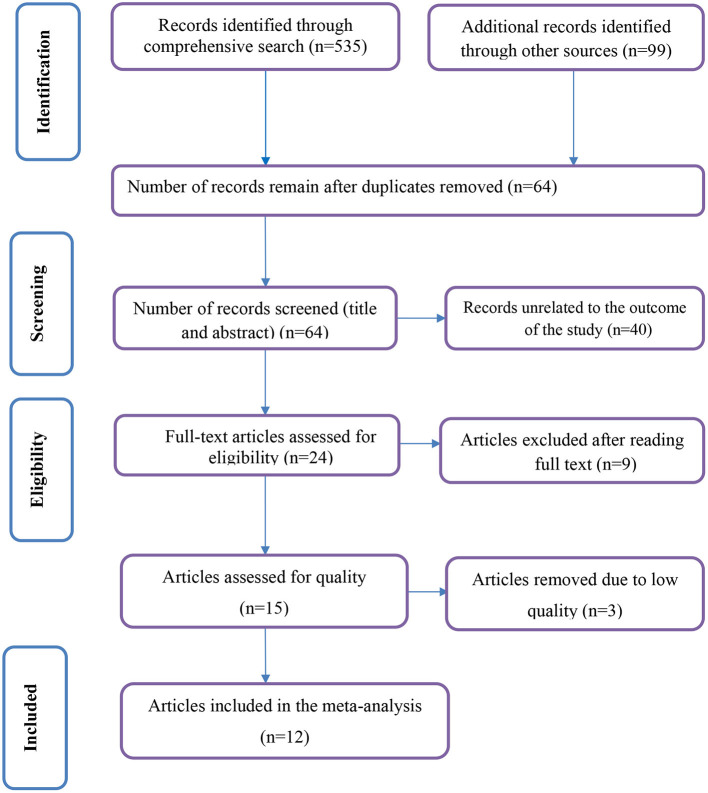
PRISMA flow diagram of included studies in the systematic review and meta-analysis.

Our search was restricted to articles published in the English language between 1st January 2010 and February 30th, 2022 in the electronic databases of PubMed, Web of Science, and Excerpta Medica databaseE. In addition, Google, Google Scholar, and the African Journal of online were searched. Through systematic and manual searching, 634 primary articles were found. Due to duplication, 570 articles were removed. The remaining 64 were screened based on their title and abstract, with 40 beings eliminated as unrelated to our study. Finally, 24 full-text primary articles were evaluated against eligibility criteria, and 12 were selected for quantitative analysis (Figure 1).

### Study characteristics

**Figure 2:**
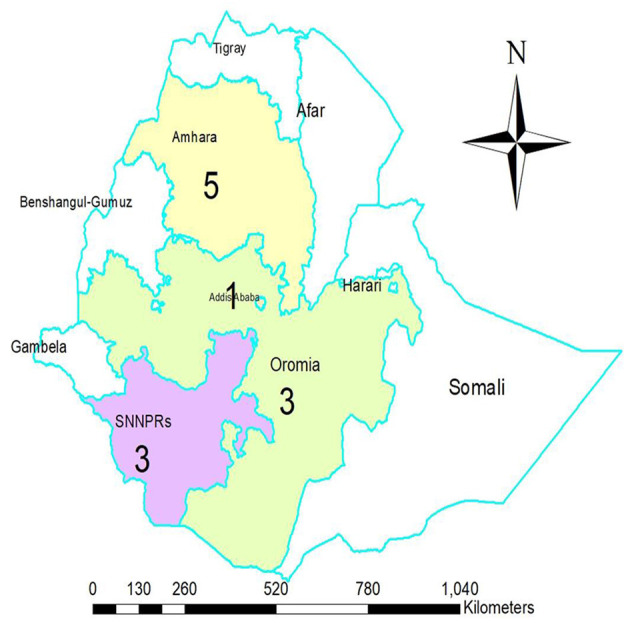
The map displays regions or city council where the primary studies included in the quantitative synthesis were conducted. (Composed by Woldegeorgis BZ. *Via* ArcGIS 10.4.1,2015 esri).

A total of 12 studies with 6,373 study participants for knowledge and 10 studies with a total of 5,336 study participants for attitudes toward epilepsy were included in this systematic review and meta-analysis. The sample size of the primary studies included in the review was significantly variable and ranged from (*n* = 135) (28) to (*n* = 840) (30) participants. Among the primary studies that reported gender (27–34, 36, 37, 41), ~3,434 (55.45%) of the participants were male. All studies employed cross-sectional research designs (27–37, 41). Of the primary studies included, three were conducted in Ethiopia's Southern Nations Nationalities Peoples' Regional State (SNNPRs) (27, 30, 37), five in Amhara Region State (29, 32–34, 36), another three in Oromia Regional State (31, 35, 41), and one in Addis Ababa, the nation's capital (28) (Figure 2).

In terms of the participants' category, eight studies were conducted among the general public or the community (27, 29–31, 33, 36, 37, 41). The remaining primary studies included school teachers (28, 32), people with epilepsy (35), and high school students (34). In the majority of primary studies (27, 29, 31–34, 36, 37, 41), representative study subjects were chosen using probability sampling techniques; two studies used consecutive participant counts (28, 30); however, one study did not mention the sampling methods that were employed (35). In addition, except in one study, which used focus group discussion on top of the interview (30), a self-administered or interviewer-administered survey tool was used to collect relevant data (27–29, 31–37, 41). To determine the level of good knowledge and favorable attitudes toward epilepsy, these primary studies utilized a yes or no response, Likert scale, modified Kilifi epilepsy beliefs, and attitude scale. The highest, 73.0%, and lowest, 12.2%, levels of good knowledge of epilepsy were reported in studies from SNNPRs, Gedeo by Molla et al. (27) and Oromia Regional State, Jimma University Specialized Hospital by Kassie et al. (35), respectively.

Furthermore, ten of the twelve primary studies (27–36) that assessed attitudes reported the highest, 74% (28), and lowest, 13% (30), levels of favorable attitudes toward epilepsy, respectively. As to the study period, all included primary studies were conducted from 2014 to 2022 with response rates ranging from 94 to 100%. Studies that had a low risk during the quality assessment were all included in this review (Table 1).

**Table 1:** The characteristics of the studies included in the systematic review and meta-analysis.

**Authors**	**Year**	**Region**	**Setting**	**Study design**	**Sampling methods**	**Sample size**	**Level of good knowledge**	**Level of favorable attitudes**	**Response rate**
Molla et al. (27)	2021	SNNPR	Gedeo	Cross sectional	Multistage random sampling	732	73%	48.4%	97.0%
Asnakew et al. (33)	2020	Amhara	South Gondar	Cross sectional	Multistage random sampling	782	33.8%	33%	96.1%
Teferi and Shewangizaw (31)	2015	Oromia	sululta	Cross sectional	Multistage random sampling	660	59.8%	35.6%	96.8%
legesse et al. (37)	2022	SNNPR	Debub bench	Cross sectional	Multistage random Sampling	601	55.1%	Not reported	96.3%
Wubetu et al. (29)	2020	Amhara	Debre Berhan	Cross sectional	Systematic random sampling	596	56.4%	58.7%	98.0%
Henok et al. (30)	2017	SNNPR	Bench-Maji	Cross sectional	Purposive sampling	840	14.3%	13.2%	99.3%
Zeleke et al. (36)	2018	Amhara	Goncha Siso	Cross sectional	Stratified random sampling	600	52.5%	65.7%	94.6%
Negussie and Geleta (41)	2018	Oromia	Jimma	Cross sectional	Systematic random sampling	300	58.3%	Not reported	100%
Oumer et al. (32)	2020	Amhara	Lay-Armachiho	Cross sectional	Cluster random sampling	568	52.8%	52.1%	97.8%
Berhe et al. (28)	2017	Addis Ababa	Addis Ababa	Cross sectional	Consecutive counts	135	53.5%	74%	94.0%
Kassie et al. (35)	2014	Oromia	JUSH	Cross sectional	Not reported	180	12.2%	70%	100%
Ferede et al. (34)	2019	Amhara	Gonder	Cross sectional	Stratified random sampling	379	47.2%	64.1%	96.0%

SNNPRs-Southern Nations Nationalities Peoples' Regional state; JUSH-Jimma University Specialized Hospital.

### Level of knowledge and attitudes toward epilepsy

**Figure 3:**
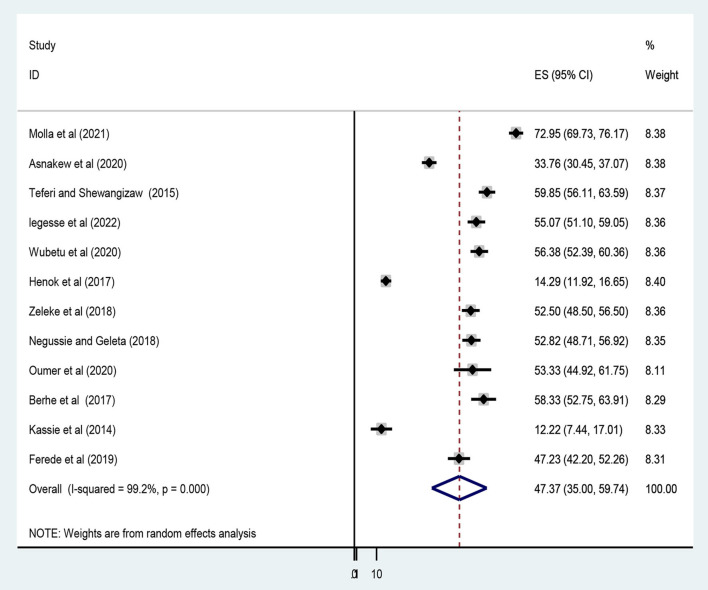
Forest plot showing the pooled prevalence of a good epilepsy knowledge among Ethiopians.

A meta-analysis was performed on 12 studies that reported a level of epilepsy knowledge and 10 studies that reported attitudes toward epilepsy. Given the substantial statistical heterogeneity in the fixed-effects model, the pooled estimate was determined using a random-effects model. Thus, an overall pooled prevalence of good level of epilepsy knowledge was only 47.37% [(95% CI: 35.00, 59.74), *I*^2^ = 99.2, *P* < 0.001] (Figure 3).

**Figure 4:**
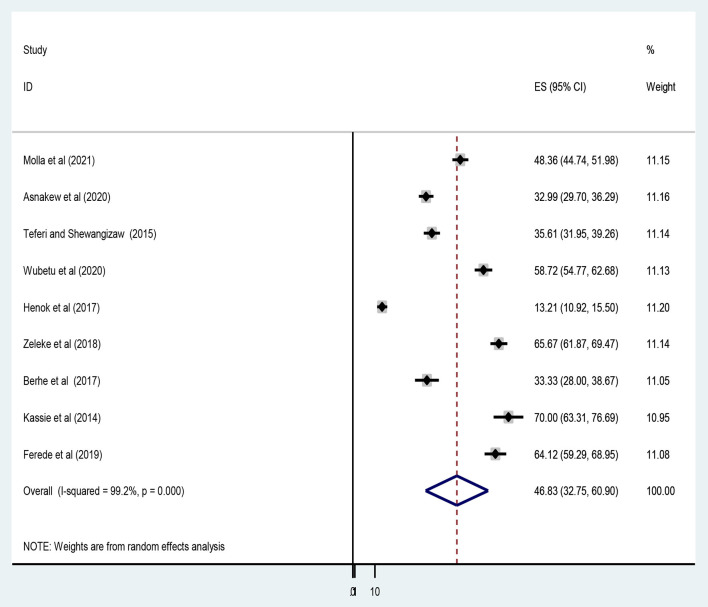
Forest plot showing the pooled level of favorable attitudes toward epilepsy among Ethiopians.

Similarly, an overall pooled prevalence of favorable attitudes toward epilepsy among Ethiopians was only 46.83% [(95% CI: 32.75, 60.90), *I*^2^ = 99.2, *P* < 0.001] (Figure 4).

### Subgroup meta-analysis

**Figure 5:**
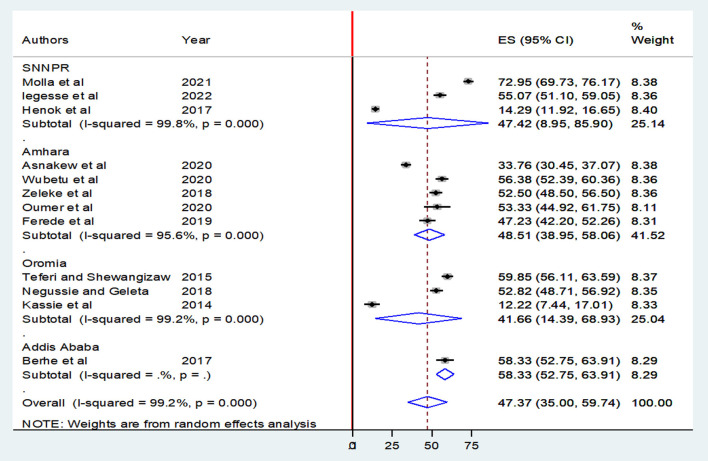
Forest plot showing subgroup meta-analysis by region for the overall prevalence of good epilepsy knowledge among Ethiopians.

Subgroup analysis was conducted based on study regions due to the presence of marked heterogeneity. Thus, the level of good knowledge of epilepsy in the Amhara region was 48.51% [(95% CI: 38.95, 58.06), *I*^2^ = 95.6%, *P* < 0.001], followed by Oromia, 41.66% [(95% CI: 14.39, 68.93), *I*^2^ = 99.2%, *P* < 0.001] and SNNPRs, 47.42% [(95% CI: 8.95, 85.90), *I*^2^ = 99.8%, *P* < 0.001] (Figure 5).

**Figure 6:**
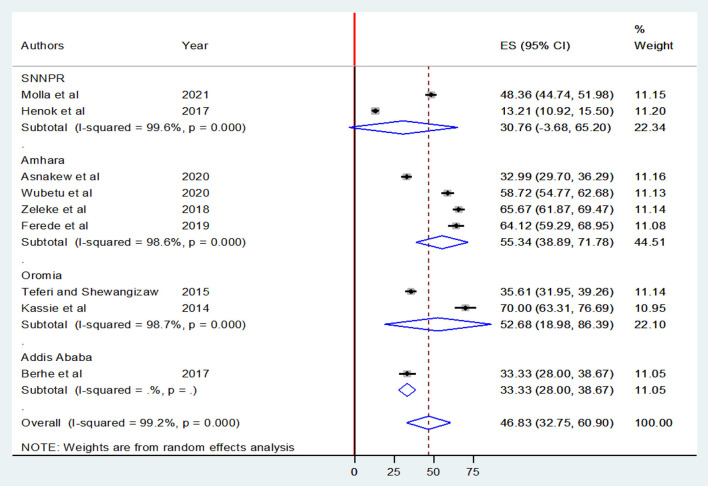
Forest plot showing a subgroup meta-analysis by region for the overall prevalence of favorable attitudes toward epilepsy among Ethiopians.

Furthermore, the pooled prevalence of favorable attitudes in Amhara, Oromia, and SNNPRs was 55.34 [(95% CI: 38.89, 71.78), *I*^2^ = 98.6%, *P* < 0.001], 52.68 [(95% CI: 18.98, 86.39), *I*^2^ = 98.7%, *P* < 0.001], and 30.76% [(95% CI: 3.68, 65.20), *I*^2^ = 99.6%, *P* < 0.001] respectively (Figure 6).

### Meta-regression

Random-effects meta-regression using sample size and year of publication as covariates was performed to explore the source of heterogeneity at a 5% significance level. As shown in Table 2, these covariates were not found to be the source of heterogeneity.

**Table 2:** Meta regression analysis of factors affecting study heterogeneity.

**Heterogeneity source**	**Coefficient**	**Standard error**	***t***	***P* > *t***	**95% confidence interval**	
**Level of good epilepsy knowledge**						
Sample size	−0.0063702	0.0239116	−0.27	0.796	−0.06044621	0.0477216
Year of publication	3.895946	2.299045	1.69	0.124	−1.304855	9.096747
**Level of favorable attitude toward epilepsy**						
Sample size	−0.0652294	0.0287732	−2.27	0.058	−0.1332672	0.0028084
Year of publication	3.141156	2.763573	1.14	0.293	−3.393656	9.675968

### Sensitivity meta-analyses

A leave-out-one sensitivity analysis was conducted to assess the impact of each study on the pooled level of favorable attitudes and a good level of knowledge regarding epilepsy while gradually excluding each study. Results showed that the combined level of good knowledge and favorable attitude did not significantly change as a result of the excluded study (Table 3).

**Table 3:** Sensitivity analysis of pooled prevalence with each study removed one by one.

**Study omitted**	**Estimate**	**95% confidence interval**	
**Good knowledge**			
Molla et al. (27)	45.024105	33.220181	56.828033
Asnakew et al. (33)	48.618805	34.996983	62.240623
Teferi and Shewangizaw (31)	46.234299	33.027714	59.440884
legesse et al. (37)	46.671364	33.306721	60.036007
Wubetu et al. (29)	46.552593	33.232292	59.872894
Henok et al. (30)	50.411606	40.792881	60.030331
Zeleke et al. (36)	46.906414	33.474293	60.338539
Negussie and Geleta (41)	46.877651	33.478882	60.276421
Oumer et al. (32)	46.845901	33.825302	59.866501
Berhe et al. (28)	46.382019	33.287518	59.476524
Kassie et al. (35)	50.562191	38.108547	63.015839
Ferede et al. (34)	47.386425	34.085598	60.687248
Combined	47.372136	35.003512	59.740761
**Favorable attitude**			
Molla et al. (27)	46.641052	30.795803	62.486298
Asnakew et al. (33)	48.573112	32.332233	64.813995
Teferi and Shewangizaw (31)	48.24185932	32.179947	64.303772
Wubetu et al. (29)	45.335846	30.325153	60.346542
Henok et al. (30)	51.023033	40.69265	61.35342
Zeleke et al. (36)	44.454826	30.306118	58.603535
Oumer et al. (32)	46.825359	32.753712	60.897007
Berhe et al. (28)	48.507038	33.071438	63.942635
Kassie et al. (35)	43.974228	29.3979	58.550556
Ferede et al. (34)	44.6679	30.002783	59.333019
Combined	46.82536	32.753714	60.897007

### Publication bias (reporting bias)

**Figure 7:**
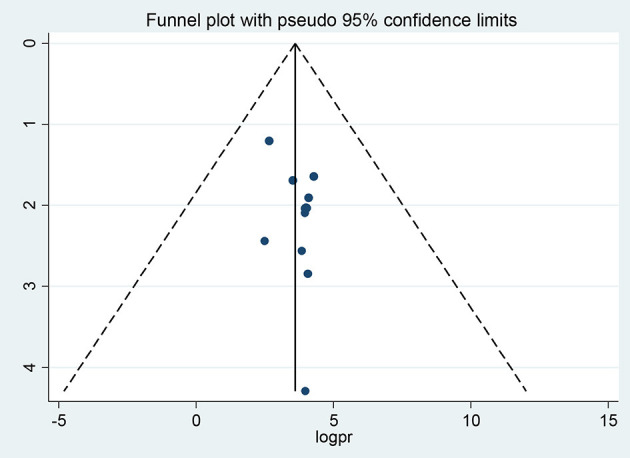
Funnel plots of publication bias for a good level of epilepsy knowledge.

Publication bias was assessed subjectively using a funnel plot and objectively by the regression-based test of Egger and the non-parametric rank correlation test of Begg at *P* < 0.05. A funnel plot showed some asymmetrical distribution (Figure 7), however, neither Egger's linear regression test (*t* = 1.55, *P* = 0.152) nor Begg's rank correlation test (*z* = 1.17, *P* = 0.244) was statistically significant for a good level of epilepsy knowledge, corroborating that there is no evidence of small study effects.

**Figure 8:**
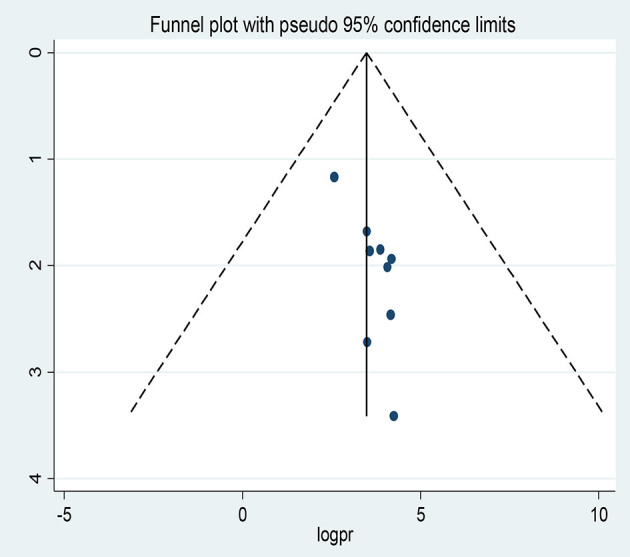
Funnel plots of publication bias for favorable level attitudes toward epilepsy.

Regarding the favorable level of attitudes toward epilepsy the visual inspection of the funnel plot showed an asymmetrical distribution (Figure 8).

**Figure 9:**
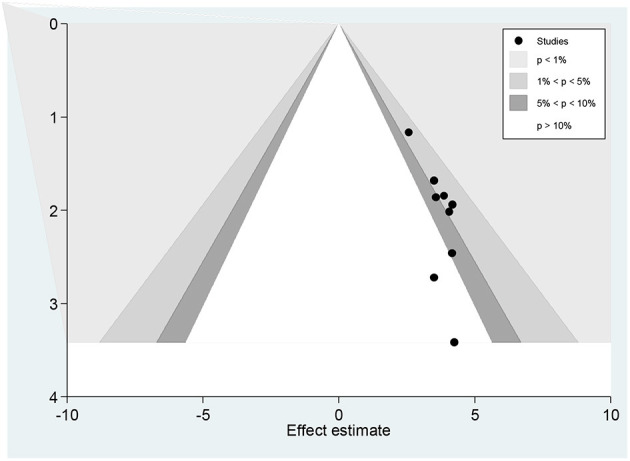
Counter-enhanced funnel plots of publication bias for favorable attitude toward epilepsy.

Moreover, the counter-enhanced funnel plot (Figure 9) showed that small studies were found in non-statistical significance (white area). So, the asymmetry may have been caused by the publication bias.

**Figure 10:**
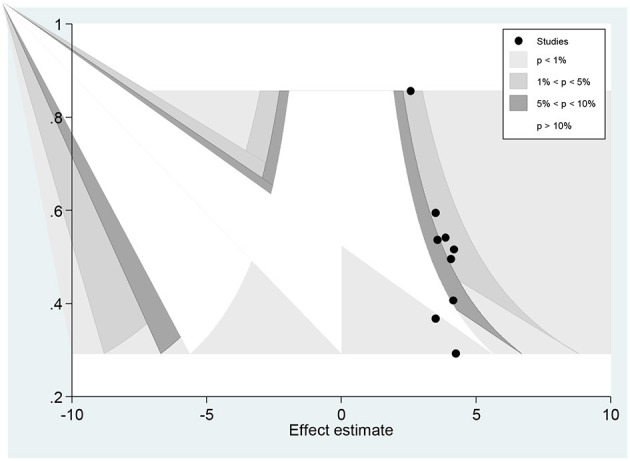
Meric inverse counter enhanced funnel plots of publication bias for favorable attitude toward epilepsy.

Similar findings were also observed when we performed the metric inverse counter-enhanced funnel plot (Figure 10).

**Figure 11:**
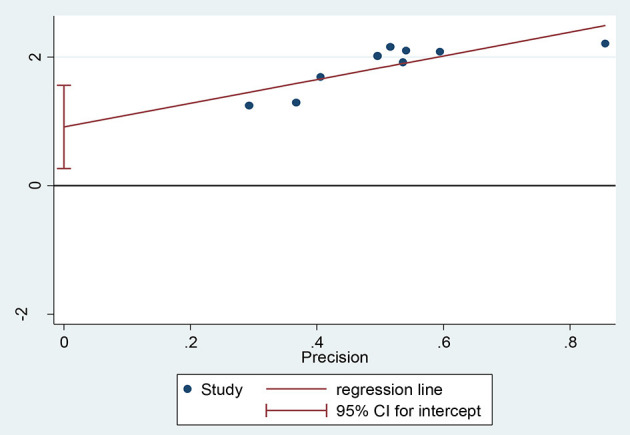
Regression graph of favorable attitude toward epilepsy.

When objectively evaluated against the Egger© regression test, the estimated bias coefficient (intercept) was 0.91385 with a standard error of 0.2732 giving a *P*-value of 0.012 and 95% CI (0.68–1.56). The test thus provides strong evidence for the presence of a small study effect. In addition, as shown in Figure 11 while only two points just touch the regression line the majority of the points were above the regression line.

**Figure 12:**
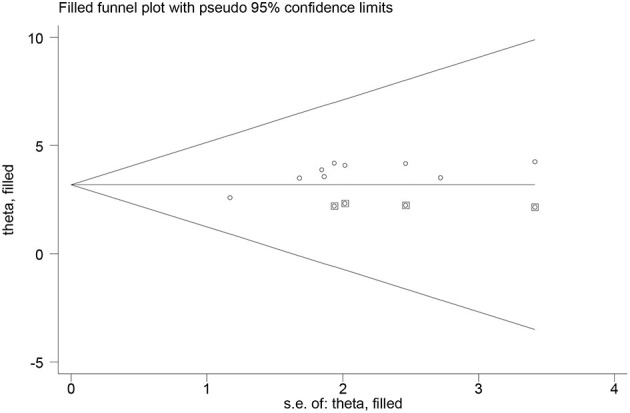
Trim and fill analysis for the prevalence of favorable attitude toward epilepsy.

Furthermore, we conducted the non-parametric trim-and-fill method of Duval and Tweedie, tests for funnel-plot asymmetry, which provides a way to assess the impact of missing studies because of publication bias on the meta-analysis. Thus, the trim and fill (metatrim) analysis showed the presence of four unpublished studies. Considering these studies in calculating the pooled prevalence yields, an estimated pooled prevalence of favorable attitude, which is adjusted for publication bias was found to be 29.74% [95% CI (14.70, 44.79), *P* < 0.001] (Figure 12).

## Discussion

To begin with, epilepsy is a common but widely misunderstood disease that primarily affects low and middle-income countries, particularly Ethiopia, a multi-cultural and multi-ethnic country. As a result, PWE faces a considerable stigma in society. Thus, good epilepsy knowledge is an important factor in reducing discrimination and negative attitudes toward epilepsy. According to the report by Tedrus et al. (42) people who lack good knowledge about epilepsy have unfavorable attitudes toward epilepsy. This review aimed to estimate the percentages of good epilepsy knowledge, and favorable attitudes toward epilepsy, and identify the associated factors. These results have been obtained from research conducted in various administrative regions of Ethiopia. According to the result of this random effect meta-analysis, the pooled prevalence of good epilepsy knowledge among Ethiopians was only 47.37% (35.00–59.74). This result indicates that more than an average of Ethiopians are not knowledgeable about epilepsy and this was by far lower than previous studies conducted in Pakistan (77.5%) (43), South Korea (94%) (44), Italy 94%, Indonesia, and Croatia (97%) (45, 46), Cameroon (99.3%) (47), and among school teachers in Egypt (100%) (48). These discrepancies could be due to variability in the study population, sample size, study period, beliefs, culture, and ethnic background which affect knowledge about the disease.

On the other hand, the pooled level of good knowledge in this review is higher than in studies in Thailand (4.6%) (49), Nigeria (15.3%), and the 2000 United States population survey (25%) (50). In subgroup analyses, in the nation's capital, Addis Ababa, the pooled prevalence of good level of epilepsy knowledge was, 58.33% (52.75, 63.91) which was relatively higher than studies in Ethiopia's Oromia, 41.66% (14.39, 68.93), Amhara region, 48.51% (38.95, 58.06), and SNNPR's regions, 47.42% (8.95, 85.90). This could be due to public media being more accessible and the majority of health professionals being centrally located.

The goal of this systematic review and meta-analysis was also to estimate Ethiopians' attitudes toward epilepsy. Misconceptions and social misunderstandings about epilepsy may have a greater impact on the quality of life of patients than the seizure itself (51). Several studies have attempted to describe that the presence of misconceptions about epilepsy, such as epilepsy being untreatable, contagious, or a form of mental retardation, (43, 49, 52–56), “mad pig disease” (57), appear to aggravate the level of unfavorable attitudes.

Moreover, epilepsy is also known as “Gila Babi” in the local Malay language, despite its use being less common today. The phrase translates to “pig insanity” because “Gila” means insanity and “Babi” means pig (58). Chinese people have referred to similar characteristics as “goat or sheep insanity” (59). Such misconceptions about insanity or mental illness and epilepsy have been widely reported in Ethiopian studies that epilepsy is caused by evil spirits, or “setan” (it means devil in the local Amharic language); that it is contagious, or that it is a form of insanity (41, 60); hereditary, or a curse from God (41).

Regarding attitudes, the overall pooled prevalence of favorable attitudes toward epilepsy was only 46.83% ( 32.75, 60.90), which indicates that a significant proportion of Ethiopians have unfavorable attitudes toward epilepsy. The findings of the current review are significantly lower than studies in Cameron: South West region, (70.6%) (61), and North West region, (77.2%) (47); India, (77.7%) (62) Trinidad and Tobago, (93%) (63). This disparity could be attributed to differences in education, population composition, methodologies, and geographical variation, as well as a strong cultural perception of the disease. On the other hand, few studies have demonstrated findings lower than our study. To mention, the first report from the Population-based, epidemiological field laboratory in the BaVi (EPIBAVI) district of the Ha Tay province, Vietnam, by Tuan et al. (64) reported that only about 33% of the residents had favorable attitudes toward epilepsy. Furthermore, in studies conducted in Egypt, only 8% of participants had a favorable attitude (65). However, there were almost consistent reports of low levels of favorable attitudes in Ethiopia studies across its regions, ranging from 45% in a study conducted in Goncha Siso Enesie Woreda Rural Kebeles, East Gojjam, Amhara region (36) to 51% in the Menit community in Benchi-Maji Zone, SNNPR (30) corroborating that there were no significant differences in epilepsy attitudes across Ethiopia's geographical regions.

Furthermore previous primary studies reported factors influencing knowledge about and attitudes toward epilepsy.Thus, Abate et al. (29) found that having completed primary school, being married, not having witnessed a seizure, and not having heard about epilepsy were all associated with a low level of epilepsy knowledge. The authors also mentioned in their study that 1,000 birr monthly income was an independent predictor of unfavorable attitude toward epilepsy in addition to these factors associated with a poor level of knowledge of epilepsy.

Individuals who did not attend modern education were more likely to have poor knowledge than those who completed high grades, according to Abate and his colleagues. The findings of this Ethiopian study were consistent with those of studies conducted in South India (66), and Ghana (67).

## Strengths and limitations of the study

This study avoided duplication of similar work because the protocol for it was registered. A double-blinded comprehensive search was conducted over a reputable period in more than seven online databases to avoid missing published studies. In addition, more than two data abstractors were involved, and to ensure inter-rater agreement, we consulted the Cochrane handbook for systematic reviews. The newly amended JBI critical appraisal tool was used for quality assessment. Further analyses were conducted to explore sources of dissemination or publication biases. We followed the updated 2020 PRISMA checklist to compile the report. Furthermore, ArcGIS was employed to locate the number of primary studies in respective Ethiopian administrative regions. The limitations of this systematic review have also been acknowledged. One of the drawbacks was the skewed distribution of studies across Ethiopia's administrative regions. Because the majority of the studies included in the current meta-analysis were conducted in three Ethiopian regions: Amhara, Oromia, and SNNPRs. As a result, it may partly affect the pooled estimates as there may be sociocultural differences across regions within a country. However, the majority of the Ethiopian population resides in these three regions; therefore, the results can locate the policy interventions that should be taken to improve knowledge about and attitudes toward epilepsy. Furthermore, the results of this review should be interpreted with caution due to significant heterogeneity in pooled effect estimates. The determinant factors meta-analysis was not pooled due to limited studies that investigated factors associated with good levels of epilepsy knowledge as well as favorable levels of attitudes toward epilepsy.

## Conclusion and recommendations

The pooled random effect meta-analysis revealed a significant knowledge and attitude gap regarding epilepsy among Ethiopians. Furthermore, previous studies have identified some of the factors such as residence, occupation, wealth index, and level of education as important and these may contribute to these gap in knowledge about and attitudes toward epilepsy. Therefore,we recommend that large-scale community and institutional-based educational intervention packages targeting different segments of the population be implemented in all Ethiopian administrative regions and city councils to reduce existing epilepsy knowledge and attitudes gaps through policy revision and engagement of local and international stakeholders.

## Author contributions

BW conceptualized the study, developed and registered the study protocol, and searched and screened articles. MO and BW were involved in data abstraction, statistical analysis, interpretation, and writing the initial and final drafts of the manuscript. EB, TK, NG, and BY were involved in the risk of bias assessment. EA, HA, HT, TB, DD, and NG contributed to the statistical analysis and writing-up of the manuscript draft. All authors contributed to the article and approved the submitted version.
