# Supplementary material for: Ethiopians' knowledge of and attitudes toward epilepsy: A systematic review and meta-analysis
Source: Front Neurol. 2023 Feb 28;14:1086622. doi: 10.3389/fneur.2023.1086622 (PMC10011168; doi:10.3389/fneur.2023.1086622)
Supplement: Supplementary file 2 [file Table_2.docx]

Table 2: concept map for the study Ethiopian knowledge of and attitudes toward epilepsy

| **Concept map 1:**Knowledge | Keywords | Knowledge[Text Word] |
| --- | --- | --- |
|  | MeSH terms | "Knowledge"[Mesh] |
| **Concept map 2:**Attitude | Keywords | "Attitude"[text word] |
|  | MeSH terms | "Attitude"[Mesh] |
| **Concept map 3:**Epilepsy | Keywords | "Epilepsy"(text word) OR "Neurologic Manifestations"[text word] |
|  | MeSH terms | "epilepsy"[MeSH Terms] |
| **Concept map 4:**Ethiopians | Keywords | Ethiopia (text word) |
|  | MeSH terms | "Ethiopia"[Mesh] |

((((Knowledge) OR ("Knowledge"[Mesh])) AND (("Attitude"[text word]) OR ("Attitude"[Mesh]))) AND (("Epilepsy"(text word) OR "Neurologic Manifestations"[text word]) OR ("epilepsy"[MeSH Terms]))) AND (("Ethiopia"[Mesh]) OR (Ethiopia* (text word)))
