# Supplementary material for: Ethiopians' knowledge of and attitudes toward epilepsy: A systematic review and meta-analysis
Source: Front Neurol. 2023 Feb 28;14:1086622. doi: 10.3389/fneur.2023.1086622 (PMC10011168; doi:10.3389/fneur.2023.1086622)
Supplement: Supplementary file 3 [file Table_3.docx]

Table 3: Quality assessment of studies using JBI’s critical appraisal tools designed for Descriptive cross-sectional study

| Study | Sample size | JBI’s critical appraisal questions | | | | | | | | | Score | Overall Appraisal |
| --- | --- | --- | --- | --- | --- | --- | --- | --- | --- | --- | --- | --- |
|  |  | Q1 | Q2 | Q3 | Q4 | Q5 | Q6 | Q7 | Q8 | Q9 |  |  |
| Molla et al | 732 | Y | Y | Y | Y | y | Y | Y | Y | Y | 9 | Included |
| Andualem H et al | 846 | Y | Y | Y | Y | Y | Y | Y | U | Y | 9 | Included |
| T. Berhe et al | 3550 | Y | Y | Y | Y | Y | Y | Y | Y | Y | 9 | Included |
| Teferi et al | 660 | Y | Y | Y | Y | Y | Y | Y | Y | Y | 9 | Included |
| Geleta et al | 300 | Y | Y | Y | Y | Y | Y | Y | Y | Y | 9 | Included |
| Kassei et al | 180 | Y | Y | Y | Y | Y | Y | Y | Y | Y | 9 | Included |
| A.Belete et al | 814 | Y | Y | Y | Y | Y | Y | Y | Y | Y | 9 | Included |
| Ferede et al | 395 | Y | Y | Y | Y | Y | Y | Y | Y | Y | 9 | Included |

Y –Yes;N-No;U -Unclear-Question. Overall score is calculated by counting the number of Y’s in each row.Q1=Was the sample frame appropriate to address the target population? Q2=Were study participants sampled in an appropriate way? Q3=Was the sample size adequate? Q4=Were the study subjects and the setting described in detail? Q5=Was the data analysis conducted with sufficient coverage of the identified sample? Q6=Were valid methods used for the identification of the condition? Q7=Was the condition measured in a standard, reliable way for all participants? Q8=Was there appropriate statistical analysis? Q9=Was the response rate adequate, and if not, was the low response rate managed appropriately?
