# Supplementary material for: Ethiopians' knowledge of and attitudes toward epilepsy: A systematic review and meta-analysis
Source: Front Neurol. 2023 Feb 28;14:1086622. doi: 10.3389/fneur.2023.1086622 (PMC10011168; doi:10.3389/fneur.2023.1086622)
Supplement: Supplementary file 4 [file Table_4.docx]

Table 4: Quality assessment of studies using JBI’s critical appraisal tools designed for Analytical cross-sectional study

| Study | Sample size | JBI’s critical appraisal questions | | | | | | | | Score | Overall Appraisal |
| --- | --- | --- | --- | --- | --- | --- | --- | --- | --- | --- | --- |
|  |  | Q1 | Q2 | Q3 | Q4 | Q5 | Q6 | Q7 | Q8 |  |  |
| Abate Dargie Wubetu et al | 596 | Y | y | y | y | y | y | y | y | 8 | Included |
| Zeleke et al | 634 | y | y | y | y | y | y | y | y | 8 | Included |
| Legesse E et al | 624 | y | y | y | y | y | y | y | y | 8 | Included |
| M. Oumer et al | 590 | Y | y | y | y | y | y | y | y | 8 | Included |

Y –Yes;N-No;U -Unclear-Question. Overall score is calculated by counting the number of Y’s in

For analytical cross-sectional study, the JBI checklist assessed the following questions Q1= were the criteria for inclusion in the sample clearly defined? Q2=were the study subjects and the=Were objective, standard criteria used for measurement of the condition? Q5=Were confounding factors identified? Q6=Were strategies to deal with confounding factors stated? Q7=Were the outcomes measured in a valid and reliable way? Q8=Was appropriate statistical analysis used?
